# Pre-Saccadic Shifts of Visual Attention

**DOI:** 10.1371/journal.pone.0045670

**Published:** 2012-09-21

**Authors:** William J. Harrison, Jason B. Mattingley, Roger W. Remington

**Affiliations:** 1 The School of Psychology, The University of Queensland, St Lucia, Queensland, Australia; 2 Queensland Brain Institute, The University of Queensland, St Lucia, Queensland, Australia; University of California, Davis, United States of America

## Abstract

The locations of visual objects to which we attend are initially mapped in a retinotopic frame of reference. Because each saccade results in a shift of images on the retina, however, the retinotopic mapping of spatial attention must be updated around the time of each eye movement. Mathôt and Theeuwes [1] recently demonstrated that a visual cue draws attention not only to the cue's current retinotopic location, but also to a location shifted in the direction of the saccade, the “future-field”. Here we asked whether retinotopic and future-field locations have special status, or whether cue-related attention benefits exist between these locations. We measured responses to targets that appeared either at the retinotopic or future-field location of a brief, non-predictive visual cue, or at various intermediate locations between them. Attentional cues facilitated performance at both the retinotopic and future-field locations for cued relative to uncued targets, as expected. Critically, this cueing effect also occurred at intermediate locations. Our results, and those reported previously [1], imply a systematic bias of attention in the direction of the saccade, independent of any predictive remapping of attention that compensates for retinal displacements of objects across saccades [2].

## Introduction

We process detail in our visual environment through a combination of shifts of spatial attention and saccadic eye movements. For spatial attention to be coordinated successfully with eye movements, the attention system must take into account the changing retinal positions of visual objects brought about by each saccade. Rolfs, Jonikaitis, Deubel, and Cavanagh [2] recently demonstrated that, just prior to a saccade, perceptual sensitivity is enhanced at a location in the opposite direction to the saccade that corresponds to the retinal location that will subserve task-relevant stimuli following the saccade. They, and others [3], have suggested these pre-saccadic changes in visual sensitivity represent the remapping of visual attention to compensate for the impending retinal displacement caused by the saccade. By contrast, a study by Mathôt and Theeuwes [1] found increases in perceptual sensitivity in the *same* direction as an impending eye movement, and argued that predictive remapping shifts the focus of attention in the direction of the saccade. In the present study, we examine further the effects described by Mathôt and Theeuwes, and conclude that shifts of attention in the direction of the saccade are probably independent of remapping mechanisms, thus explaining this apparent empirical discrepancy.

**Figure 1:**
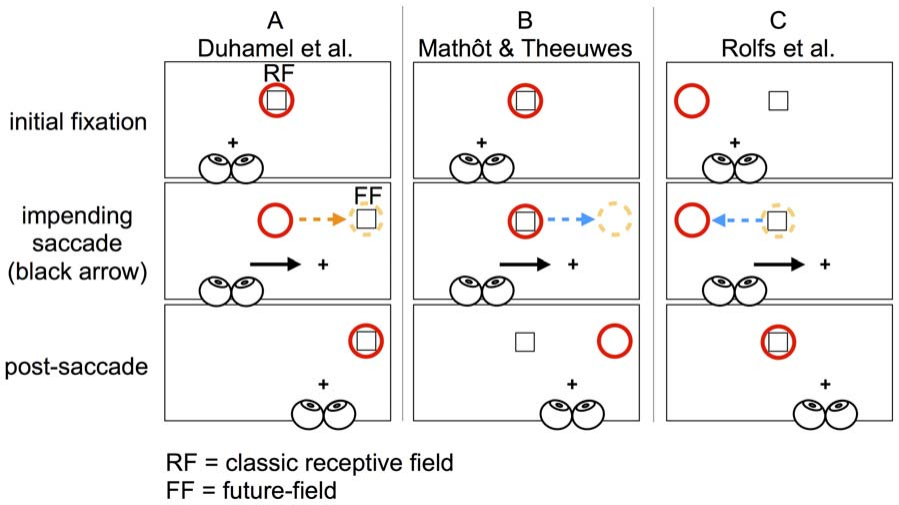
Examples of remapping and shifting attention across saccades. (A) Responsiveness of single cells prior to a saccade (e.g. [*4*]). During initial fixation (the cross), the retinotopic location of the receptive field of a population of cells is shown in red; these cells would initially respond to the onset of the square. When there is an impending saccade, however, these cells should begin to respond to a stimulus presented within their future-field (dotted orange circle). (B) Mathôt and Theeuwes [*1*] suggested attention is predictively remapped in the direction of the saccade. According to their account, if attention at the dotted circle is subserved by the same cells as in (A), attending to a cue (the square) should increase the firing rate of these cells during fixation. Because of predictive remapping, these cells should begin to respond to stimuli at the cells' future-field just prior to the saccade. As suggested by Mathôt and Theeuwes [*1*], [*8*], the cued cells' increased firing rate might enhance processing of targets within the future-field. Such a change in attention, however, could also result in attention being allocated to a non-cued location following the saccade, which would be functionally irrelevant. (C) “Functional remapping”, as demonstrated by Rolfs et al [*2*]. Remapping attention in this case is subserved by a population of neurons similar to those shown in (A), but whose classic receptive field is to the left of fixation. Because the position of the cue will shift from the right visual field during fixation to the left visual field following a rightward saccade, functional remapping of attention would require a shift of attention in the opposite direction to the saccade. This attention shift compensates for the retinal shift of visual objects following the saccade.

The behaviour of single cells throughout the visual attention system has provided an insight into how retinotopically mapped visual attention may be maintained across saccades. Brain activity in areas associated with saccade generation and spatial attention suggests that neurons in these regions predict the retinal consequences of eye movements. For example, as shown in Figure 1a, Duhamel, Colby, and Goldberg [4] found that cells in the macaque lateral intraparietal area (LIP) begin to respond to a stimulus outside the cells' receptive field when an impending saccade brings the stimulus into the receptive field. That is, these cells begin to respond to the predicted post-saccadic location of the receptive field, hereafter called the “future-field”. This change in activity around the time of a saccade is referred to as remapping [4]. Cells showing a remapping response have been found in other regions also involved in attentional control, including the frontal eye fields [5] and superior colliculus [6]. These changes in neural activity may represent the updating of a target's location on a retinotopic salience map, guiding the deployment of spatial attention to task relevant information across saccades [3]. Importantly, just prior to a saccade, the responses of remapping cells anticipate a stimulus appearing within the receptive field following the saccade.

**Figure 2:**
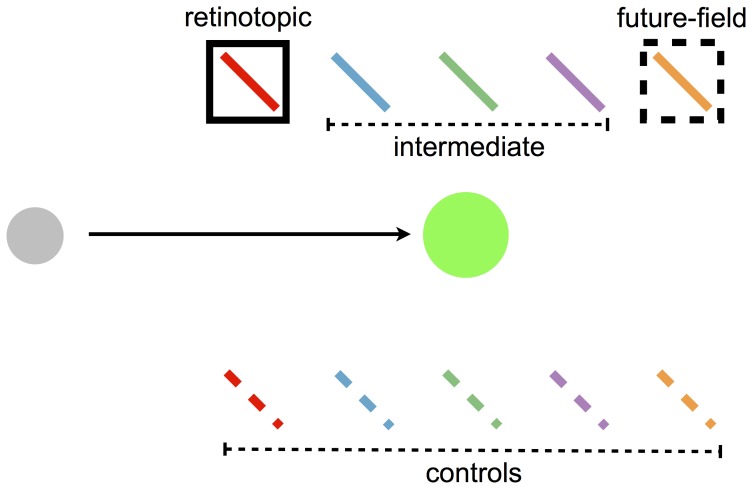
The basic arrangement of stimuli employed in the current experiments, adapted from Mathôt and Theeuwes [**1**]. Participants made a saccade from a grey spot to a green spot, as indicated by the arrow. Prior to the saccade, a cue (the black square) was briefly flashed. Shortly following the offset of the cue and still prior to the saccade, a target was shown at locations represented by the tilted bars. In Mathôt and Theeuwes' study, the target appeared at the retinotopic or future-field location of the cue, or one of their relative control locations (the broken tilted bars). In the present study, we also probed several intermediate locations.

Recently, Mathôt and Theeuwes [1] examined how shifts of spatial attention are coordinated with eye movements, and argued that pre-saccadic shifts of spatial attention are similar to the change in responsiveness of remapping neurons (see Figures 1a and 1b). Mathôt and Theeuwes combined a standard spatial cueing paradigm (e.g. [7]) with an eye movement task. They had participants make a saccade either horizontally or vertically to the location of a visual marker. Just prior to the saccade, a non-predictive cue briefly appeared midway between the initial fixation point and the saccade goal, offset 45° above or below the required saccade trajectory. After the cue disappeared, but prior to the saccade, a target (a tilted bar) was presented at one of four locations: the retinotopic location of the cue; the “future-field” location (the display location corresponding to where the cued region of the retina would fall following the eye movement); or one of two uncued, “control” locations. Control locations were distant from the retinotopic or future-field locations, but matched for retinal eccentricity (see Figures 1 and 2). After executing the saccade, participants made speeded responses to the orientation of the target.

Relative to the uncued locations, participants responded faster to targets presented at the retinotopic location of the cue and at the future-field location of the cue. Mathôt and Theeuwes [1] interpreted their results as showing that visual attention, captured at the retinotopic location of the cue, was partially remapped to the future-field of the cued location, thus facilitating responses to targets at both these locations. They further speculated on the underlying neurophysiology of their results, arguing that the cue excited a population of remapping neurons during the pre-saccadic interval, so that processing of targets presented in the neurons' future-field was facilitated due to an increase in their baseline firing rate (see also [8]).

Although Mathôt and Theeuwes' [1] interpretation of their data appears consistent with the neurophysiological remapping findings (compare panels a and b in Figure 1), their interpretation raises a concern: a shift of attention in the direction of the saccade, in the manner outlined in Figure 1b, seemingly serves no functional purpose [2]. That is, a pre-saccadic shift of the focus of attention from the cued location to a location that is in the direction of the saccade results in attention being deployed to a location that was important neither prior to nor following the saccade. As argued by Rolfs et al., and as shown in Figure 1c, for attention to be allocated to the cued location following an eye movement, it must shift in the *opposite* direction to the saccade. Rolfs et al. refer to this shift of attention as “functional remapping”. In this hypothesis, a population of remapping neurons whose receptive fields fall at the predicted post-saccadic location of the cue would show the classic anticipatory remapping response [2], [4]. Furthermore, Mathôt and Theeuwes' suggestion that the focus of attention is shifted in the direction of the saccade via predictive remapping is inconsistent with the notion that remapping neurons anticipate a stimulus falling within their receptive field following the saccade [3], [4].

These issues raise the question of whether Mathôt and Theeuwes' [1] findings do in fact represent remapping of spatial information to compensate for retinal displacements of visual objects following a saccade. For example, although Mathôt and Theeuwes ruled out the possibility that their result could be accounted for by the cue and target appearing in the same visual quadrant (see [9]), they did not rule out the possibility that attention spreads more generally in parallel to the saccade vector *across* visual quadrants. Hughes and Zimba [10] demonstrated that, when cued along an oblique visual meridian, visual attention does indeed spread across the visual quadrants during fixation. If attentional benefits occurred *between* the retinotopic and future-field locations of the cue in Mathôt and Theeuwes' paradigm, spreading of attention [10] could account for their data without invoking predictive remapping.

In the current study, we asked whether the shifts of attention observed by Mathôt and Theeuwes [1] are unique to the retinotopic and future-field locations of an attended cue, or whether the cue also affects the perception of stimuli between these locations. We followed Mathôt and Theeuwes' experimental design by presenting cues and targets during the pre-saccadic interval. In addition to probing the retinotopic and future-field locations of the cue, however, we also probed intervening locations (the “intermediate” area in Figure 2). As discussed in a recent review [8], if attention is remapped predictively as a consequence of relevant cells discretely shifting the spatial location to which they respond, we might expect the focus of attention also to shift discretely from the retinotopic coordinates of an attended location to that location's future-field prior to the saccade. Alternatively, however, if attention spreads more generally, intermediate locations should also receive some attentional benefit. In two experiments we found evidence for a cueing effect at both the retinotopic and future-field locations, replicating Mathôt and Theeuwes [1]. Crucially, however, we also found significant effects of attention at intermediate locations.

In an initial experiment, we probed for attentional facilitation at intermediate locations between the retinotopic and future-field locations of a brief cue flashed just prior to a saccade. If attention to the cue location shifts directly from the retinotopic coordinates of the cue to future-field coordinates, then perception should be facilitated at these locations, but not at intermediate target locations (see Figure 2). By contrast, if attentional benefits of the cue apply more generally across space, targets presented at intermediate locations should be responded to faster than (uncued) control locations.

## Methods

### Ethics statement

Prior to testing, each participant was given an information sheet outlining what was required of him or her during the experiment. Participants were informed via the information sheet and verbally by the experimenter that they were free to withdraw from the experiment at any time without penalty. Before testing began, informed consent was obtained verbally from all those who participated in the study. Verbal consent was deemed sufficient due to the experiment not involving any foreseeable risk beyond that of everyday living. The study and consent procedure were approved by The University of Queensland's School of Psychology Ethical Review Committee (code: 09-PSYCH-PhD-38-CVH).

### Participants

Twenty-nine (29) individuals from The University of Queensland participated in Experiment 1 for a monetary reward (AU$10) or course credit. Participants were aged from 18–42 years (*M* = 23.15, *SD* = 3.70; 12 females), and all were naive to the purpose of the experiment with the exception of one of the authors (WJH). All participants reported normal or corrected to normal vision.

### Materials

Participants sat with their head in a head and chin rest positioned 57 cm from an LCD monitor (60 Hz). Stimuli were generated using Presentation (Neurobehavioral Systems). Eye movements were recorded at 500 Hz with an EyeLink 1000 (SR Research, Canada).

### Stimuli and procedure

**Figure 3:**
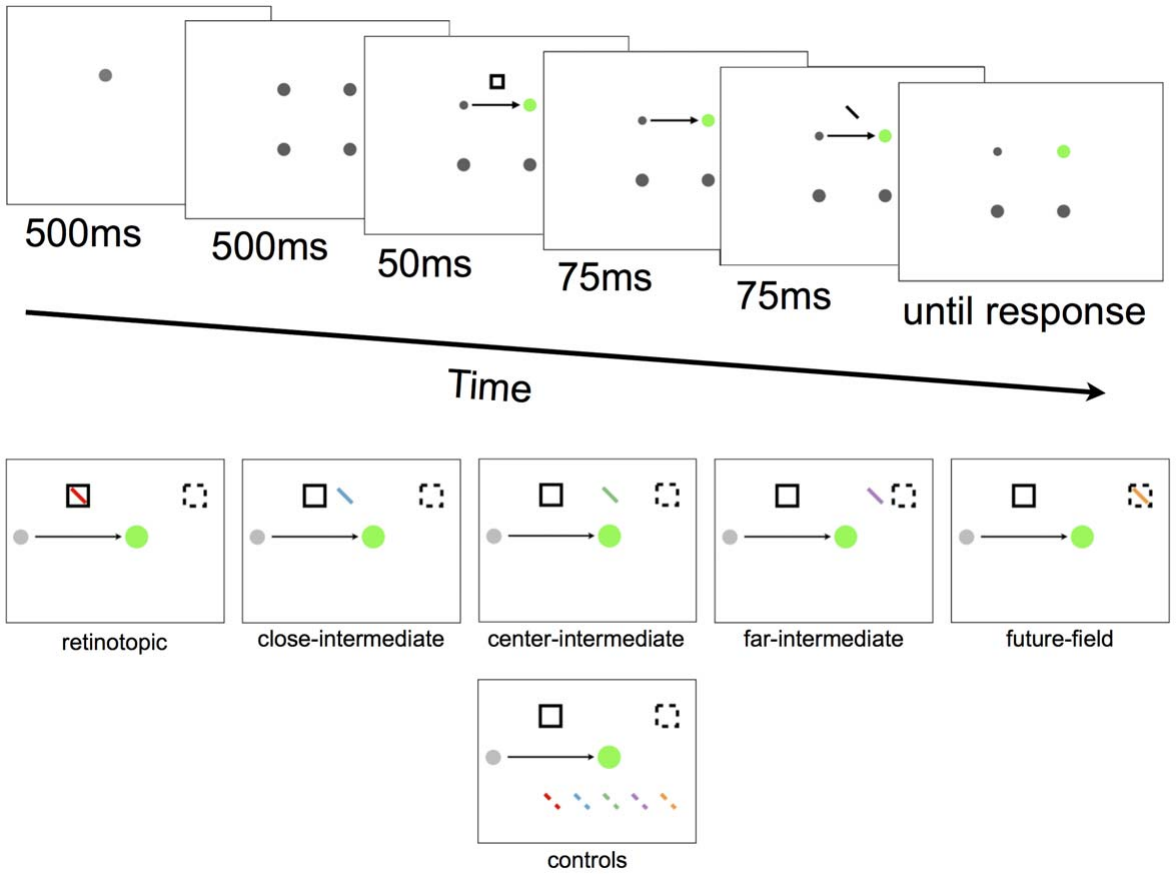
Example trial sequence from Experiment 1 (top) and all tested target locations (bottom). In this example, the participant first fixates the top left spot for the first two frames and is then required to make a saccade towards the green spot at the same time as the onset of the cue (square frame). In this example, the target (the tilted bar) appears at the retinotopic location of the cue, but can actually appear at any target location shown in the bottom panel. Note there was only one target presented per trial, all targets and cues were grey, and the background of the display was black.

The display and procedure were the same as those described by Mathôt and Theeuwes [1], except that extra target locations were included. Figure 3 shows the displays in a typical trial sequence. In each trial, participants were required to make a saccade and then to respond to the orientation of a tilted bar. Each trial began when participants fixated a single grey spot (1.5°) presented at one of four possible locations. After 500 ms, three more grey spots were presented, forming a square of 9°×9°. After a further 500 ms, a single grey spot, aligned horizontally or vertically with fixation, turned green to signal the saccade target. At the same time, the fixation spot reduced in size, and the visual cue (a square frame of 1.8°×1.8°) was flashed for 50 ms. The cue could appear at one of two possible locations that deviated from the required saccade trajectory by −45° or 45°, and was located 6.4° of visual angle from the fixation and saccade target spots. Participants were instructed to make an eye movement to the green spot as quickly and as accurately as possible.

The target was a 2.5° bar with a width of 0.25° presented 125 ms after the onset of the cue, for a duration of 75 ms. The bar was tilted 45° left or right off vertical. There were 10 possible target locations relative to the direction of the required eye movement (see Figure 2 and Figure 3). The tilted bar could appear at the retinotopic location of the cue, or at the future-field location of the cue. In addition, targets could appear at any of three locations between the retinotopic and future-field locations (intermediate locations). The distance between adjacent target locations was 1.8°. We refer to the retinotopic, future-field and intermediate target locations as “test” locations. Targets could also appear at five “control” locations that were directly opposite the test locations (see Figure 3). The target appeared at each location with equal probability irrespective of the cue location (i.e., the cues were not predictive of target location). Participants were instructed to report the orientation of the bar as quickly as possible (tilted left or right) by pressing a left or right arrow key. Auditory feedback signalling a correct or incorrect response was provided, and the screen then went blank for 1500 ms before the next trial began. Participants completed two practice blocks, the first consisting of 10 trials of just the saccade task, and the second consisting of 20 full trials. There were 32 trials for each target location, resulting in 320 experimental trials per participant.

## Results

### Data filtering

We excluded trials using the same screening criteria as Mathôt and Theeuwes [1]. Data from three participants who had more than 30% of their trials excluded were omitted from analyses. Data from a further two participants who had error rates higher than 20% in a single condition, and from a single participant for whom the eye-tracker could not be calibrated reliably, were also omitted from analyses. For the remaining participants, trials were removed if gaze deviated by more then 2° from the initial fixation point (0.5% of trials), if the saccade trajectory deviated more than 22.5° from the saccade target (6.2% of trials), if saccade latency was below 100 ms or above 600 ms (1.1% of trials), if response time was below 200 ms or above 1000 ms (3.4% of trials), or if a participant's gaze arrived at the saccade target before the offset of the target (1.8% of trials). In total, 86.9% of trials from 23 participants were included in the statistical analyses.

### Response times

As shown in Figure 3, response times were faster to targets presented at the retinotopic, future-field, and at two of the three intermediate locations than their relative controls. We conducted a repeated measures ANOVA with the factors condition (test and control) and location (retinotopic, close-intermediate, center-intermediate, far-intermediate, and future-field; see Figure 3). There were significant main effects of both condition, *F*(1, 22) = 12.96, *p* = .002, and location, *F*(4, 88) = 3.99, *p* = .005, but no significant interaction, *F*(4, 88)<1.

To assess whether the cue facilitated responses to targets at different locations, we conducted planned comparisons on mean response times to targets at test versus control positions at each of the five spatial locations. Response times were significantly faster to targets at both the retinotopic and the future-field locations than their control locations, *t*(22) = 2.09, *p* = 0.049, and *t*(22) = 2.34, *p* = 0.029, respectively, thus replicating the findings of Mathôt and Theeuwes [1]. Response times were also significantly faster when the target was presented at the far-intermediate location compared with its control location, *t*(22) = 2.25, *p* = 0.035, and marginally faster when the target was presented at the center-intermediate location compared with its control location, *t*(22) = 1.97, *p* = 0.062. There was no difference in response times to targets at the close-intermediate target location and its control location, *t*(22) = 0.44, *p* = 0.662.

**Figure 4:**
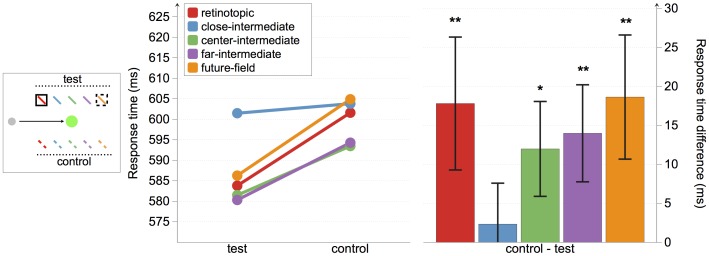
Experiment 1 response-time results. The left panel depicts absolute response times for all conditions. The right panel shows the response time differences between test and control locations, with positive values indicating faster responses to targets at test than control locations. Error bars represent standard error. Asterisks indicate that differences are different from 0. *p<0.10; **p<0.05.

In summary, we found a significant cueing effect at both the retinotopic and the future-field locations, as expected. Crucially, we also found evidence for a cueing effect at two out of three intermediate locations (see Figure 4).

There is a possibility that our exclusion criteria, based on Mathôt and Theeuwes [1], failed to eliminate inaccurate saccades. Because the intended saccade goal determines which screen positions correspond to the future-field of the cue, “intermediate” targets may have been processed preferentially when saccades were inaccurate. If this had occurred, it might have generated a spurious cueing effect at one or more of the intermediate locations. We therefore re-analysed the data including only saccades that fell within 2° of the saccade target. This resulted in the exclusion of a further 0.3% of trials only, demonstrating that participants were generally highly accurate in the saccade task. As for the response time data, this more stringent analysis again yielded significant differences in response times at all test locations compared with their control locations (all *p*'s<0.05), with the exception of the close-intermediate location.

### Errors and saccadic latencies

Planned comparisons revealed no differences in the frequency of target errors between the test and control locations (all *p*'s>0.15). Nor was there any such difference for saccadic latencies (all *p*'s>0.20; Table 1).

**Table 1:** Experiment 1 error rates and saccadic latencies (ms).

	retinotopic	close-intermediate	center-intermediate	far-intermediate	future-field
**Errors**					
test	4%	4%	4%	5%	4%
control	5%	4%	4%	4%	4%
difference	1%	0%	0%	−1%	0%
**Saccadic latencies**					
test	228	227	224	223	228
control	224	229	226	224	227
difference	−4	1	2	1	−1

## Discussion

The aim of Experiment 1 was to determine whether, just prior to a saccade, attention is allocated exclusively to the retinotopic and future-field locations of an exogenous cue. Participants responded faster to targets at the retinotopic and future-field locations of the cue, and at the far-intermediate location. Responses to targets at the center-intermediate location fell just short of being significantly different from responses to targets at a control location, but when we restricted analyses to trials showing high-precision saccades, this difference became significant.

We offer two explanations for finding a cueing effect at only two out of the three intermediate locations. First, in support of the notion of a pre-saccadic spread of attention, the lack of a cueing effect at the close-intermediate location could be due to attentional suppression around the cue [11], [12]. Previous studies have demonstrated a “suppressive annulus” surrounding an attended location that spans approximately 2° [11], [12]. Only the close-intermediate probe location fell within this region. Second, in support of the notion that attention shifts discretely from retinotopic to future-field locations, the influence of the cue at two out of the three intermediate locations could be due to those locations' proximity to the saccade endpoint. It is well established that during saccade planning attention shifts to the goal of the saccade (e.g. [13], [14]). An interaction between attention shifting towards the saccade goal and towards the future-field location could account for our finding at the close- and far-intermediate target locations. Therefore, in Experiment 2, we changed the design of the experiment and probed a single, intermediate location that should be free from both of these issues.

### Experiment 2

**Figure 5:**
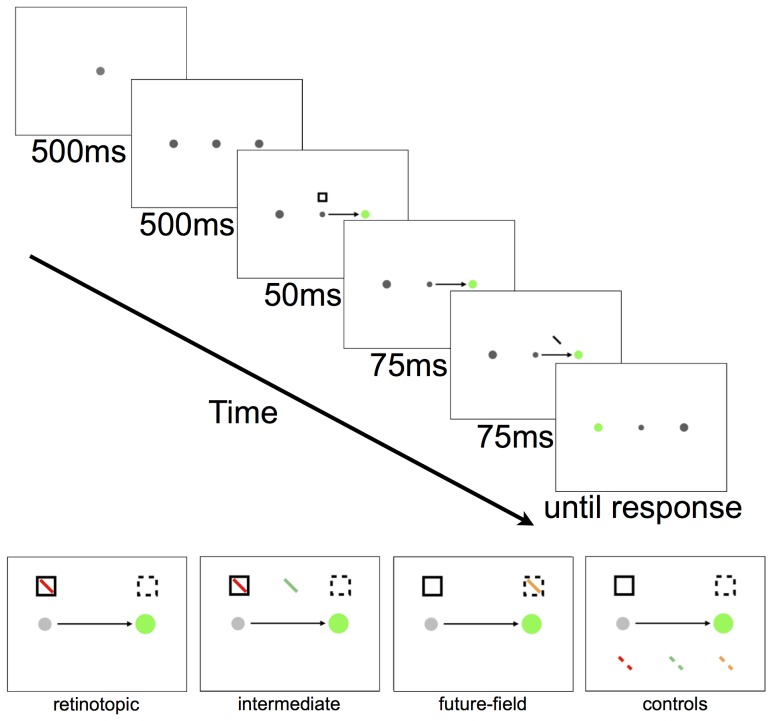
Example trial sequence from Experiment 2 (top) and all tested target locations (bottom). In this example, the participant first fixates the center spot for the first two frames and is then required to make a saccade rightwards to the green spot at the same time as the onset of the cue (square frame). The target (the tilted bar) appears at the intermediate location. All target locations are shown in the bottom panel.

In Experiment 2 we replicated the cueing effects observed at intermediate locations in Experiment 1 using a design that was free from the two potential spatial limitations mentioned above. We aimed to probe an intermediate location that should be unaffected by any attentional suppression around the cue, and that should not be preferentially processed based on its proximity to the saccade goal. The changed design can be seen in Figure 5. To prevent attentional suppression around the cue location from affecting responses to targets adjacent to the cue [11], [12], we chose a single intermediate target location beyond the region expected to be suppressed, and equidistant from the retinotopic and future-field locations. We also changed the display configuration so that the saccade goal could not influence target processing at the intermediate location. If attention shifts directly from retinotopic to future-field coordinates, there should be no facilitation of responses to targets presented at the intermediate target location. Alternatively, if attention spreads between these locations, any cueing effect should be equivalent across retinotopic, future-field, and intermediate target locations.

### Method

#### Participants

Nine individuals from The University of Queensland participated in Experiment 2. They ranged in age from 17–27 years (*M* = 21.22, *SD* = 4.24; 4 females), and received a monetary reward (AU$10) or course credit. All were naive to the purpose of the experiment with the exception of WJH, and all had normal or corrected to normal vision.

#### Materials

These were the same as in Experiment 1.

#### Stimuli and procedure

Figure 5 shows an example trial sequence from Experiment 2. Equipment and testing conditions were the same as in Experiment 1. Each trial began with a fixation spot at the center of the screen for 500 ms. Two additional spots were then presented for 500 ms along the horizontal meridian, at 9° to the left and right of fixation. One of the two peripheral spots then turned green to signal the saccade target, and the fixation spot reduced in size. At the onset of the saccade target, the cue appeared for 50 ms at 3.8° above or below the fixation spot.

The target was presented for 75 ms, 125 ms after the onset of the cue, at one of six locations for each possible eye movement (leftward or rightward). As in Experiment 1, the target appeared either at the retinotopic location of the cue, or at the future-field location of the cue. Due to the change of location of the cue in Experiment 2, the retinotopic and future-field target locations were now adjacent to the start- and end-point of the saccade, respectively. Targets were also presented at a location equidistant (2.8°) from the retinotopic and future-field target locations (the intermediate location). This intermediate location was not only distant from the saccade goal, but also distant from the cue location, reducing the possibility that targets at this location could be affected by any attentional suppression around the cue [11]. There were also three control locations, matched for the retinal eccentricities of the retinotopic, future-field, and intermediate locations. Targets were presented at each location with equal probability, making the cue location non-predictive of target location, as in Experiment 1. Responses, auditory feedback and intertrial intervals were also the same as described in Experiment 1. Participants completed 20 practice trials before commencing 480 experimental trials, yielding 80 trials per target location.

### Results

#### Data filtering

Using the same criteria as in Experiment 1, trials were excluded on the basis of gaze deviation (<0.1%), saccade deviation (2.4%), saccade latency (0.6%), response time (1.0%), and when participants' eyes arrived at the saccade target before the offset of the probe target (1.0%). In total, 94.9% of trials from nine participants were included in the analysis.

#### Response Times

**Figure 6:**
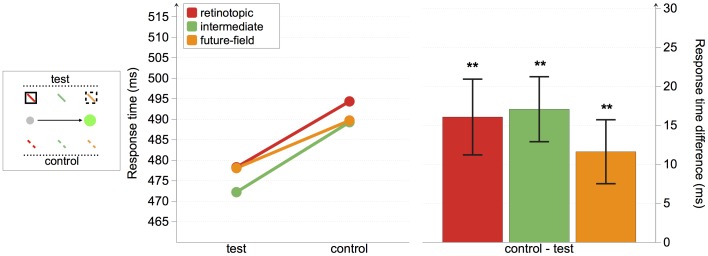
Experiment 2 response-time results. The left panel depicts absolute response times for all conditions. The right panel shows the response time differences between test and control locations, with positive values indicating faster responses to targets at test than control locations. Asterisks indicate that differences are different from 0. **p<0.05.

As shown in Figure 6, participants responded faster to targets presented at the retinotopic, future-field, and intermediate locations compared with their control locations. We conducted a repeated-measures ANOVA with the factors condition (test and control) and location (retinotopic, intermediate, and future-field). We found a significant main effect of condition, *F*(1, 8) = 22.13, *p* = .002, but no main effect of location, *F*(2, 16) = 1.05, and no interaction, *F*(2, 16)<1.

Planned comparisons revealed that response times were significantly faster for targets presented at both the retinotopic and the future-field locations of the cue, compared with their respective control locations, *t*(8) = 3.31, *p* = 0.011, and *t*(8) = 2.83, *p* = 0.022, respectively. These cueing effects replicate those from Experiment 1, and are also consistent with the findings of Mathôt and Theeuwes [1]. Crucially, there was also a significant cueing effect when the target was presented at the intermediate target location, equidistant from the retinotopic and future-field locations, *t*(8) = 4.09, *p* = 0.003.

#### Errors and saccadic latencies

Error rates for Experiment 2 are shown in Table 2. Planned comparisons conducted on error rates revealed no differences between any of the test locations and their control locations (all *p*'s>0.091).

**Table 2:** Experiment 2 error rates.

	retinotopic	intermediate	future-field
**Errors**			
test	3%	5%	5%
control	6%	5%	5%
difference	3%	0%	1%

**Figure 7:**
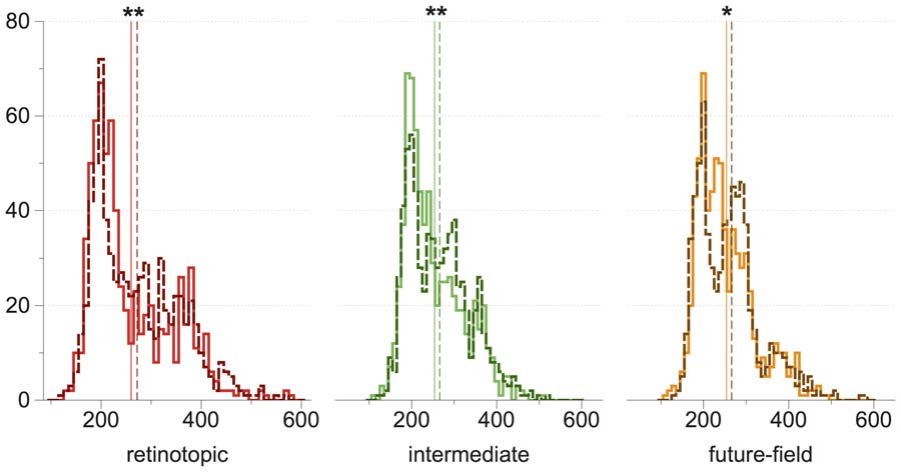
Experiment 2 saccadic latency distributions. Frequencies are plotted on the y-axis, and mean saccadic latencies are plotted on the x-axis (vertical lines). Color codes correspond to those used in Figure 6, with solid lines representing test conditions and dotted lines representing control conditions. Asterisks indicate significant differences between means. *p<0.10; **p<0.05.

Saccadic latency distributions and their means are plotted in Figure 7. Generally, saccadic latencies were shorter when targets appeared at the test locations than when they appeared at the control locations (see Figure 7). Planned comparisons confirmed that saccade latencies were shorter when targets were presented at the retinotopic and intermediate locations compared with their controls, *t*(8) = 2.79, *p* = 0.024, and *t*(8) = 2.58, *p* = 0.033, respectively. Latencies were marginally shorter when targets were presented at the future-field location compared with its control, *t*(8) = 2.23, *p* = 0.056.

#### Response time analysis according to probe-saccade onset asynchrony

**Figure 8:**
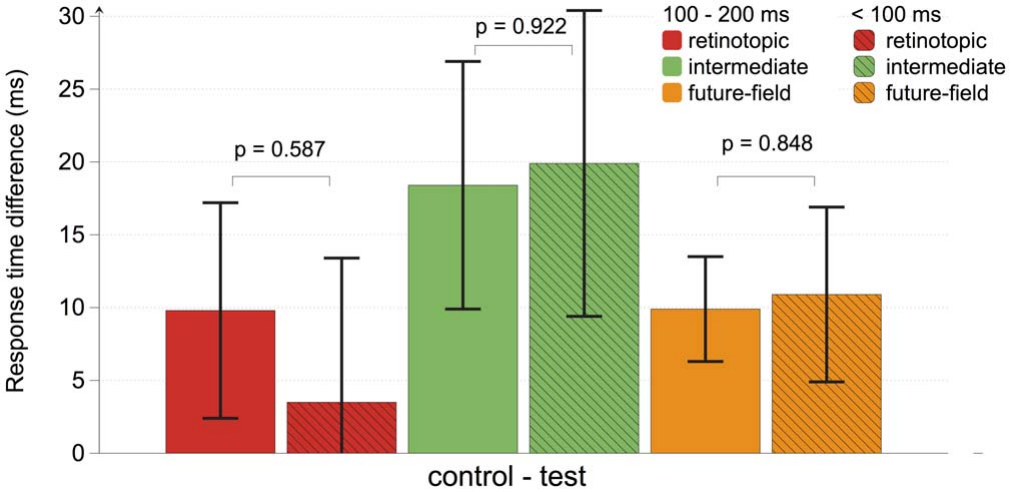
Experiment 2 response-time analyses according to probe-saccade onset asynchrony. According to probe-saccade onset asynchrony, trials were sorted into 100 ms time bins. Response time differences between test and control locations were then calculated for each condition at each time interval, with positive values indicating faster responses to targets at test than control locations. No changes in response times across time bins were found.

Predictive remapping alters responses of single neurons within approximately 100 ms of saccade onset, without significantly affecting neuronal activity at longer intervals prior to the saccade [4]–[6], [15]. To examine whether our results conform to this established pattern we binned trials according to the onset time of the probe relative to saccade onset on a trial-by-trial basis. We classified probe onsets as occurring either within 100 ms of saccade onset, or between 100 and 200 ms before saccade onset. One participant was omitted from this analysis because there was only a single trial in which the probe appeared within 100 ms of saccade onset and other trial parameters met the inclusion criteria listed above. In Figure 8, we show the cueing effect (response time difference between test and control locations) for each probe location according to relative probe timing. For each probe location, there were only small changes in the cueing effect across time. Importantly, at the future-field location, there was less than 2 ms difference in the cueing effect across time bins (all *p*'s>0.58, uncorrected). We conducted a similar analysis comparing data from the final 100 ms prior to a saccade against trials in which the probe was presented earlier than 200 ms prior to saccade, which revealed similar non-significant changes across time. These results argue against a predictive remapping account of our results, which should have shown greater modulation of response times when probes were presented within 100 ms prior to a saccade [2].

#### Saccade trajectories

**Figure 9:**
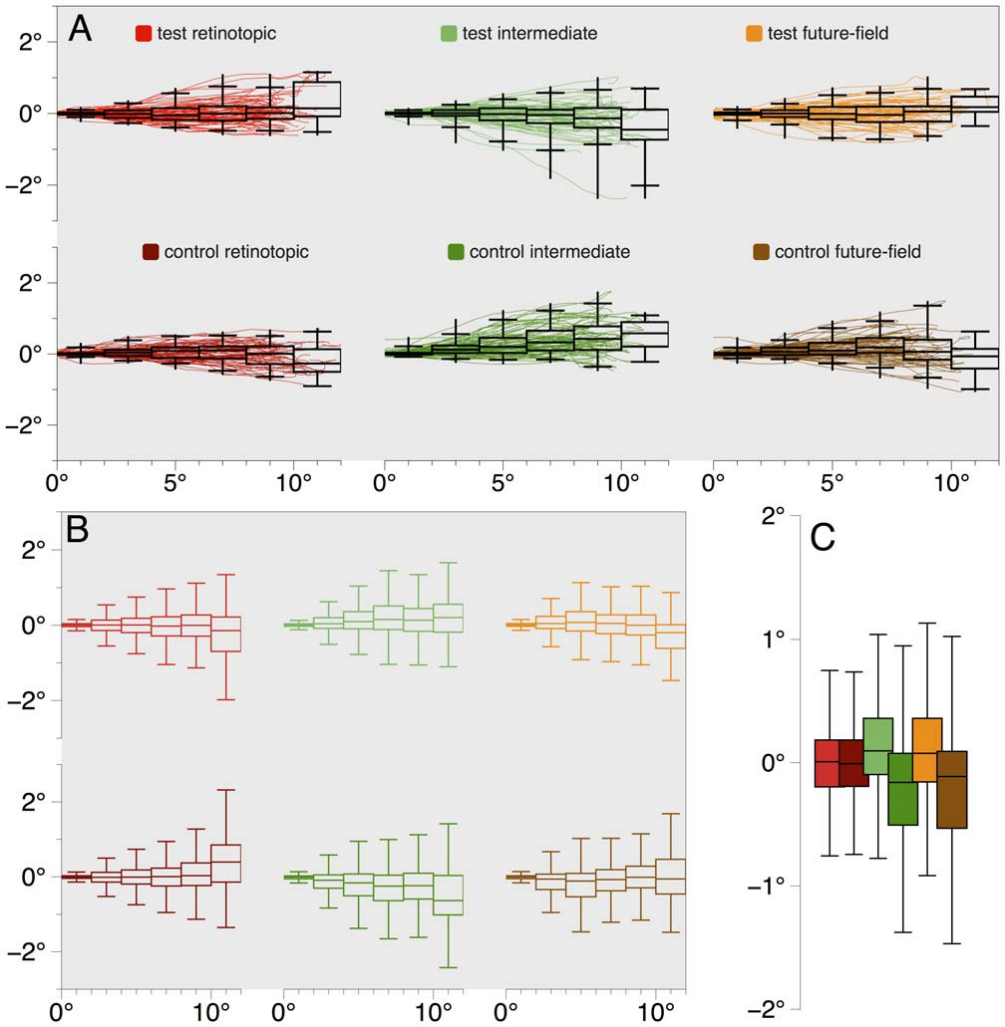
Experiment 2 saccade trajectories. (A) Saccade trajectories for a single observer. All data were normalized to show all saccades as originating from the same position (0°, 0°) and as directed rightwards, and with the cue location above the saccade starting point. This normalization procedure would exaggerate any consistent trajectory patterns. Box and whisker plots show saccadic deviation across 2° bins, and reveal there was no consistent deviation in saccade trajectories across conditions for this observer. For each bin, the central band of a box represents the median deviation, the upper and lower bounds of the box represent the 25^th^ and 75^th^ percentiles, the extent of the whiskers show the range, and the whisker terminators indicate the 2.5^th^ and 97.5^th^ percentiles. As can be seen from the median deviations over the length of saccades, across conditions there is no consistent deviation toward or away from the cue location for this observer. (B) Box and whisker plots of saccade deviations for all observers in Experiment 2 revealed a lack of reliable deviation across conditions. Box plots are as described in (A). Whiskers show 1.5× interquartile range. The apparently high variability in saccade endpoints in the 10–12° bin likely arises from the low number of saccades that overshot the target at 9° (for example, see Figure 9A), and our normalization method described above. Any deviations from a direct line were small and non-significant. (C) To test if saccade trajectories were curved, we examined deviations at half the distance of the saccade length (4–6° bin shown in Figure 9B), when deviations should be greatest [*16*]. For each condition we found only small, non-significant deviations from zero and no differences across conditions. Condition colors in (B) and (C) correspond to those shown in (A).

We analyzed saccade trajectories to determine whether the cue had any effect on saccadic programming [16]. Across conditions, we found no significant deviations from a straight line. Individual traces of saccades from a single observer (author WJH) whose response times were representative of the group are shown in Figure 9A. Average trajectory data from the group are shown in Figure 9B. All deviations were small, and were not statistically different from zero (all *p*'s>0.05, uncorrected).

### Discussion

In Experiment 2 we again found that, prior to the saccade, a non-predictive cue facilitated response times to targets presented not only at the retinotopic and future-field locations, but also at an intermediate location between them. Furthermore, an examination of response times according to when the probe was presented relative to saccade onset showed there was no change in the cueing effect at the future-field in the final 100 ms prior to a saccade, contrary to what would be expected from predictive remapping [2], [4]–[6], [15].

## General Discussion

The aim of the present study was to examine how visual processing is affected by an attentional cue presented just prior to a saccade. Mathôt & Theeuwes [1] recently demonstrated that a cue presented just prior to a saccade attracts attention not only to the cue's retinotopic location, but also to the future-field of that retinotopic location. We examined cueing effects at intermediate spatial locations, and found significant cueing effects for them as well as for retinotopic and future-field positions. The overall pattern of results suggests that response time facilitation at retinotopic and future-field locations is not unique but occurs at locations within the interval from the cue to the future-field. Mathôt and Theeuwes [1], [8] have suggested that their results are consistent with neurophysiological data showing that neurons in several regions of the visual attention system begin to respond to stimuli in their future-field just prior to a saccade (e.g. [4]). Early findings suggested that remapping neurons in FEF respond only to stimuli within their future-field and not at intermediate locations [15], but more recent data suggest some neurons in FEF respond to stimuli at intermediate locations [17], [18]. The pre-saccadic shifts of attention we observed appear broadly consistent with these more recent findings, though this hypothesis will need to be tested in future studies.

Regardless of the apparent parallels with the relevant neurophysiological findings, we suggest that these results – and those of Mathôt and Theeuwes [1] – reflect more general changes in attention allocation during the pre-saccadic interval, and that these changes are probably independent of saccadic remapping neurons. As argued by Rolfs, et al. [2], changes in activity of remapping neurons appears to anticipate a stimulus appearing in the cells' receptive-field following an eye movement, and this anticipatory change affects a location that is in the *opposite direction* to the saccade. In contrast, the probed locations in the present study (and that of Mathôt and Theeuwes) were in the same direction as the saccade. We therefore assume that the reliable attention effects we have observed must arise from the operation of mechanisms other than those evoked during predictive remapping.

As outlined in the Introduction, remapping attention in the direction of the saccade does not preserve attention in world-centered coordinates [2]. Why, then, might attention be redistributed in the manner revealed in the present study? One possibility is that, rather than serving a role in remapping per se, these effects represent disruption of the normal allocation of attention to the saccade goal (e.g. [13]). Attentional capture at the location of the cue requires that attention be subsequently reoriented toward the saccade goal before a saccade can be executed [14], thus facilitating processing of stimuli across a region of space as attention shifts from one location to another [19]. Alternatively, the response time differences observed between the retinotopic and future-field locations compared with control locations could have been due to participants responding more quickly to apparent motion [20]; faster responses might have arisen from apparent motion perception, rather than through changes in attention prior to the saccade. Such a finding would also contradict the predictive remapping account.

In replicating Mathôt and Theeuwes' [1] work, we felt it important to replicate their response-time measure. However, seminal studies examining pre-saccadic attention shifts have used masked, force-choice discrimination measures that limit the potential of post-saccadic decisions to affect responses (e.g. [13], [14]). Response time measures leave open the possibility that some facilitation of targets at the future-field location could arise from a post-saccade retinotopic trace of the cue [21]–[24]. But this account also requires an interaction with memory for the target, because the target was extinguished before the end of the saccade. We think this is unlikely. Moreover, a post-saccade memory trace of the cue cannot account for the cueing effects we observed at “intermediate” locations.

On first inspection, our findings appear inconsistent with those of Golomb et al. [23], who failed to find facilitation for intermediate cues *following* a saccade. However, there is at least one critical difference between the studies: Golomb et al. were interested in how, following an eye movement, visual attention is re-deployed to a world-referenced location defined prior to the saccade. Relative to the retinotopic location, therefore, the spatiotopic and intermediate locations they examined were in the opposite direction to the executed saccade, similar to those locations required for “functional remapping” as argued by Rolfs et al. [2] and outlined in Figure 1C. In contrast to this, we probed for pre-saccadic attention shifts in the same direction as the saccade (see Figure 1B and Figure 2). Since our intermediate positions did not correspond to those of Golomb et al., the results of the two studies are not directly comparable.

In two experiments we observed facilitation of target processing at locations between the retinotopic and future-field locations of the cue. In this regard the findings of the present experiments do not support a special status for the future-field location and, hence, question whether the results from this paradigm reflect remapping. Instead, these data suggest that an attentional cue presented in the pre-saccadic interval may affect processing of visual information across a broad region of space on the side of the saccade trajectory. The broad facilitation of response time for locations in the direction of the impending saccade may represent transitory changes in the allocation of attention when a briefly flashed cue competes for attention with the saccade target.
